# Urgency Theory in the context of broader emotion theories: a conceptual review

**DOI:** 10.3389/fpsyt.2024.1403639

**Published:** 2024-07-05

**Authors:** Lindsey Fisher-Fox, Christiana J. Prestigiacomo, Melissa A. Cyders

**Affiliations:** Department of Psychology, Indiana University Indianapolis, Indianapolis, IN, United States

**Keywords:** urgency, decision-making, emotions, adaptive, maladaptive urgency and broader emotion theories

## Abstract

Negative and positive urgency are two closely related personality traits that reflect the tendency for an individual to engage in maladaptive risk-taking in response to extreme negative and positive emotions, respectively. However, other prominent emotion theories describe how emotions contribute to adaptive, rather than maladaptive, decision-making. This conceptual review considers how Urgency Theory can be integrated with these broader existing emotion theories. We proceed as follows: a) briefly define what is meant by emotions in science and summarize basic human neuroscience underlying emotions; b) briefly describe select theories and research linking emotions to adaptive decision-making, including brain correlates of this effect; c) review Urgency Theory, including contrasting evidence that emotions lead to maladaptive outcomes and brain correlates of this effect; d) discuss how urgency can be integrated into theories that view emotions as both adaptive and maladaptive for decision-making; and e) propose future directions to advance research in this field. We identified four, not mutually exclusive, viable options to integrate Urgency Theory into existing theories: urgency as model-free emotion regulation, urgency as being driven by incidental emotions, urgency as a reflexive response to emotions, or urgency as an individual difference factor. We conclude that although all four options are viable, individual difference and model-free emotion regulation have the most empirical support to date. Importantly, the other two options are less well-researched. Direct tests comparing these integrations is necessary to determine the most accurate way to integrate urgency with existing emotion theories. We believe that this research can identify mechanisms underlying urgency and help inform future intervention and prevention development to reduce negative effects of urgency across numerous maladaptive behaviors and clinical disorders.

## Introduction

The experience of emotions is fundamentally adaptive; emotions serve to focus attention to relevant stimuli and prepare the body for action in response to such stimuli (e.g., 1–3). Because of their ubiquitous nature, emotions have long been the focus of psychological study, with research suggesting that they influence a number of explicit and implicit factors related to action, including decision-making (e.g., 4), risk/reward estimations (e.g., 5), attention (e.g., 6), persistence in goal pursuits (e.g., 7), fight vs. flight vs. freeze reactions (e.g., 8), and job performance (e.g., 9).

Cyders and Smith (10) proposed a novel integration of emotions with impulsive response, which they coined “urgency.” Key rationale for Urgency Theory is that emotions undermine adaptive decision-making (e.g., 4, 11–14), through less discriminative use of information (15–17), increased distractibility (18), and an increased focus on short-term, rather than long-term, goal pursuits (19). However, there is opposing evidence that emotions can improve, rather than undermine, decision-making. For example, mild increases in positive affect improve problem solving skills (20, 21), such as cognitive flexibility (22, 23), verbal fluency (24), and problem solving (25, 26). There are also anecdotal examples of emotions leading to adaptive behaviors, such as seeking out treatment. Integrating Urgency Theory with broader emotion theories can advance understanding of how and why urgency may contribute to maladaptive outcomes, which can be important for future intervention and prevention development.

## Emotions – key terms, definitions, and basic neuroscience

We rely on the classic conceptual framework of Russell (27) to briefly define and differentiate between constructs to be used in the remainder of this review. Core affect, in the Russell (27) framework, is a general term that encompasses both discrete emotions and more diffuse moods. Emotions are discrete experiences directed at or about a specific event, person, or situation (referred to as an object) that are usually shorter in duration, whereas moods refer to a period of prolonged diffuse affect that is not necessarily directed at a specific event, person, or object (27). Ekman (28) describes several factors that make up the experience of core affect; however, here we focus on two independent dimensions relevant to the current review, valence (i.e., pleasure to displeasure) and arousal (i.e., activated/energetic to deactivated/calm). Most discrete emotions can be placed upon these two continua. For example, high arousal and high pleasure represent elation and happiness, high arousal and high displeasure represent distress or anger, low arousal and high pleasure represent calm, and low arousal and high displeasure represent sadness or lethargy. Barrett and colleagues (29) describe the valence dimension as making up the mental representation of emotion, whereas arousal is related to the physical sensations of affect.

## Theories suggesting that emotions contribute to adaptive decision-making

Emotions help us set priorities for attention and action and can facilitate adaptive choices and action plans. Pinker (30) said it best: “Most artificial intelligence researchers believe that freely behaving robots will have to be programmed with something like emotions merely for them to know at every moment what to do next” (p. 374). This focusing of attention prepares the individual to respond to meet that emotion. For example, without emotions, we might not know whether to prioritize responding to an email (neutral emotional valence and low emotional arousal in most cases, although there surely are some exceptions to this) or calling a friend whose partner recently passed away (negative emotional valence and high emotional arousal for both the friend and the individual). The negative valence and arousal in the second situation focus attention on the most pressing need and motivates behavior toward the more salient task (and away from the more emotionally-neutral task).

Early neuroscience theories on how emotions affect decision-making focus on distinct systems for affect and cognition, with emotions localized in the limbic system (e.g., 31). A meta-analysis by Kober and colleagues (32) summarized that subcortical activations in the amygdala, ventral striatum, thalamus, hypothalamus, and periaqueductal gray are most commonly found in studies of emotion generation, with an important relationship between these subcortical regions and medial regions of the prefrontal cortex, which may play an important role in the cognitive generation of emotional states. An additional meta-analysis by Lindquist and colleagues (33) concluded that emotions are experienced in the brain through an interacting set of brain regions associated with not only emotion, but also cognition and perception. Phelps and colleagues (34) conclude that multiple neural circuits underlie how emotions influence decision-making depending on a number of contextual factors, including type of emotion or affect experienced and the type of decision-making under study (e.g., risky decisions, social decisions, etc.) (34).

Here, we briefly describe select theories documenting an adaptive role of emotions for decision-making, highlighting potential mechanisms of such an effect and levels of affect at which this effect may occur. For a broader review, see Lerner and colleagues (35).

### Somatic marker hypothesis

The Somatic Marker Hypothesis, first proposed by Damasio and colleagues (36), suggests that deficits in decision-making seen in individuals with damage to their ventromedial prefrontal cortex are due, in part, to their inability to use emotional signaling to evaluate decisional options. Damasio sees emotions as a key, functional part of adaptive decision-making, as somatic markers signal that attention is needed to a particular decision and then help one to evaluate the value of potential responses or decisions (36, 37). Negative somatic markers lead to avoidance of a particular potential response, whereas positive somatic markers lead to approach of an incentive (36, 37). Thus, when somatic markers are absent, decision-making is more random and less advantageous (36). The Somatic Marker Hypothesis proposes that the representation and regulation of emotions in the brain occurs not only in the limbic system, but also in regions of the brain thought to underlie decision-making and planning (e.g., the ventromedial prefrontal cortex, the somatosensory cortices, the basal ganglia, and the insula; 38), suggesting that emotions result from brain sensory input, and shape decision-making and planning, at a neural level (39).

### Affect heuristic theory

Affect Heuristic Theory proposes that people use affect heuristics, defined as “representations of objects and events in people’s minds that are tagged to varying degrees of affect,” to guide decision-making (40). When people make decisions, they consult their pre-existing affect heuristics, which makes decision-making more efficient (40). A recent neuroimaging study found co-activation in the left insula, left inferior frontal gyrus, and left medial frontal gyrus was inversely related to the use of an affect heuristic, suggesting that affect heuristics may be negatively related to giving into momentary affective urges (41).

### Affect-guided planning and anticipation

According to Davidson (19), adaptive emotion-based decision-making functions through emotions being linked with more adaptive goals (i.e., the anticipated positive affect, or reduction of negative affect, associated with job success) rather than less adaptive ones (i.e., the reduction of immediate stress through binge eating). Davidson (19) proposes that the prefrontal cortex is responsible for mental representation of goals; when emotions occur, they can be inconsistent with such goals (e.g., when job success is incompatible with disordered level substance use) or they can be consistent with long-term goals (which Davidson calls “affect-guided planning). When emotions are inconsistent with long-term goals, the prefrontal cortex should attempt to override signaling. Asymmetries in prefrontal cortex function appear to distinguish between those who engage in affect-guided planning and those who do not (e.g., 42, 43), suggesting that the ability to maintain anticipated emotions for adaptive pursuits especially in the face of strong, more immediate reinforcers relies on left prefrontal cortex function (19).

### Broaden and build theory

The Broaden and Build Theory (44), describes the function of positive emotions as to expand one’s thoughts and action urges (i.e., to make one’s thinking and behavior more creative, more open, and more explorative). Such expansion then allows the individual to develop new skills, resources, and relationships, which are adaptive for survival and produce more positive emotional experiences in return. In Fredrickson’s (44) theory, positive emotions are adaptive in that they broaden life experiences and build in new adaptive responding patterns. There is good evidence that positive emotions function to improve decision-making. Fredrickson (45) posits that “positive emotions transform people for the better” and promote future and psychological wellbeing, whereas negative emotions have been adaptive for survival in life threatening situations, such as fear and avoidance when encountering a large animal. A study found that positive affect predicted more flexible coping strategies, as well as future emotional wellbeing (46). In a study utilizing the broaden and build framework, Reschly and colleagues (47) found that, in a sample of high school students, positive emotions predicted greater student engagement in school and greater adaptive coping, showing that positive emotions have similar positive outcomes in adolescents as well.

### Dialectical behavior therapy and wise mind

Dialectical Behavior Therapy was developed originally as a treatment for Borderline Personality Disorder; the therapeutic focus on emotions and their ability for adaptive functioning is housed in the common experience that emotions precipitate maladaptive action or inaction (48). Dialectical Behavior Therapy focuses on the cultivation of “wise mind,” in which one can utilize and integrate their own rational thoughts (i.e., “rational mind”) and emotional reactions (i.e., “emotion mind”) to make adaptive, intuitive decisions. There is evidence of adaptiveness of emotions as integrated into Dialectical Behavior Therapy. “Wise mind” has been shown to be important for treatment engagement and success (e.g., 49, 50). Kristeller and Jordan (49) found that a mindfulness-based eating awareness training program increased wise mind, which then led to increases in spirituality, well-being, and improved overall self-regulation. Kearney and colleagues (51) suggest that a meditation intervention increased unactivated pleasant, but not activated pleasant, emotions over time, and decreased both activated and unactivated unpleasant emotions; thus, concluding that the cultivation of wise mind might facilitate both unactivated pleasant emotions and better treatment outcomes for a variety of clinical disorders.

## A review of Urgency Theory: emotions contribute to maladaptive decision-making

### Development of urgency theory

Research identifying urgency began with the appreciation that impulsivity describes multiple, separate domains of behavior (52). Whiteside and Lynam (52) took an empirical approach and factor analyzed existing impulsivity measures to identify the common, underlying factors of impulsivity. Results of their factor analysis produced four distinct impulsive traits: sensation seeking (i.e., the tendency to seek out novel and exciting experiences), lack of planning (i.e., the tendency to act without thinking), lack of perseverance (i.e., the inability to remain focused on a task), and urgency (i.e., the tendency to respond rashly in response to extreme emotional states). From these results, Whiteside and Lynam (52) created the UPPS Impulsive Behavior Scale, which has shown usefulness across various populations and has been translated into several languages (e.g., 53–56).

Research then provided evidence that urgency was not a unitary construct and was instead comprised of two separate, albeit related, constructs of negative and positive urgency (57). This led to the addition of a positive urgency subscale into a revised version of the UPPS scale (The UPPS-P Impulsive Behavior Scale; 58). This revised scale is widely used to measure impulsive personality and, like the original scale, has been shown to produce valid and reliable estimates of impulsive behavior traits across age, gender, clinical populations, and language (59–63).

Urgency Theory posits that rash action is thought to occur in response to a specific and discrete experience of emotion that is directed to a specific situation, person, or object (10). When one is experiencing emotions intensely, the loss of available cognitive resources and the interference with rational decision-making increases the likelihood that one’s actions will be ill-advised or rash (10). The exact mechanisms of how urgency imparts risk remain of debate. Billieux and colleagues (64) suggested that urgency is driven by poor ability to inhibit prepotent responses during emotional contexts. Eben and colleagues (65) suggested that negative emotions may create a discrepancy between one’s current state and desired state. Previous work suggested that urgency was not explained by the interaction of over-reactivity of emotion and lack of planning (e.g., 66). However, later neuroimaging evidence found that urgency is related to increased brain response to negative images (67). Other work suggests that urgency may be driven by over-reactivity to emotional triggers, combined with a deficient ability to regulate this response (64).

### Urgency is related to maladaptive risk-taking behaviors

Negative and positive urgency are both associated with involvement in a variety of risky behaviors (10); however, there are some unique associations with different risk-taking behavior across these traits that are important to highlight. Negative urgency is more highly related to increased drinking quantity (i.e., number of drinks consumed in a single drinking episode), drinking to cope, and development of an Alcohol Use Disorder (68, 69). Research has also shown that negative urgency is a strong predictor of severity of problems across a variety of domains including medical, employment, social, family, psychiatric, and substance and alcohol use in those with a substance use disorder (70). Additionally, negative urgency has been shown to predict problems such as pathological gambling, increased eating problems, self-harm behaviors, risky sexual behavior, compulsive shopping, and craving for cigarettes (71–75).

Positive urgency has also been linked to risky behavior. For example, research has also shown that individuals are prone to engage in heavy and high-risk drinking, pathological gambling, and binge eating when experiencing elevated positive mood states (76–78). Positive urgency uniquely predict risky drinking, risky sexual behavior, and increased drug use in first year college students compared to negative urgency (79, 80). Positive urgency is also associated with increased risk-taking behavior among children (81).

### Urgency is related to psychopathology

Urgency has been implicated as a unique risk factor for and as a characteristic of psychopathology. As Johnson and colleagues (82) concluded, “A large body of work indicates that urgency is more robustly related to psychopathology than are other forms of impulsivity … Collectively, this work is beginning to transform psychopathology research to focus on integrating these domains [negative and positive urgency].” Negative urgency has been proposed as a common transdiagnostic endophenotype for a number of ill-advised risk behaviors and clinical disorders (67) and has been associated with disordered eating, Borderline Personality Disorder symptoms, nonsuicidal self-injury, substance use disorder, and aggression (83–85).

Borderline Personality Disorder symptoms are often associated with urgency due to the emotional and impulsive nature of the disorder. Martin and colleagues (86) identified a relationship between insight and urgency within Borderline Personality Disorder: Increased levels of positive urgency were associated with increased clinical insight, meaning the more the patient experienced positive urgency, the more aware of the disorder the patient was, potentially driven by self-reflectiveness, which may have implications for treatment outcomes. Urgency also predicts a more severe course of externalizing behaviors such as earlier onset of alcohol use, alcohol dependence, and smoking cessation difficulty (87–89). Johnson and colleagues (82) assert that negative urgency predicts symptoms worsening (e.g., more drinking problems) over time during negative emotional states, while positive urgency predicts more alcohol use during positive emotional states. These effects were still present even when controlling for other forms of impulsivity or emotionality. Similarly, Howard and Khalifa (90) posit that urgency contributes to the severity and is a core feature of personality disorders and may, in turn, help to explain the link between personality disorders and violence.

Emerging research has also linked urgency with internalizing psychopathology. Research suggests that urgency predicts depression and anxiety symptoms, suicidality, and obsessive thoughts in Obsessive Compulsive Disorder with similar effect sizes between negative and positive urgency, indicating both positive and negative emotions play a strong role in psychopathology (85, 91). In addition, positive and negative urgency were found to mediate the relationship between Post-Traumatic Stress Disorder symptoms and risky behaviors (92). These patterns of associations with psychopathology have also been replicated in a sample of children and adolescents. in that urgency broadly predicts a range of psychopathology such as conduct disorder, depression, panic and anxiety, and attention deficit hyperactivity disorder (ADHD) – inattentive symptoms (93). Additionally, urgency has been studied as a link in the relationship between adult ADHD and the severity of alcohol dependence, suggesting that those with ADHD may drink to cope when struggling to regulate their emotions (94). Urgency has also been found to be associated with serious suicidal ideation in 9- and 10-year-old children and is particularly salient in White children compared to Black and other race children (95).

Urgency has been implicated in psychotic psychopathology, such as Bipolar Disorder and Schizophrenia. Johnson and colleagues (96) found that urgency was associated with self-harm, suicidal ideation, and suicide attempts with negative urgency being the strongest predictor of suicidal ideation within those with Bipolar I Disorder. The relationship between urgency and suicidal behaviors was still present when controlling for major depression and other psychopathology. Negative and positive urgency are elevated in those with remitted Bipolar I Disorder and schizophrenia (97, 98). Muhtadie and colleagues (98) found that those with Bipolar I Disorder were more likely to engage in impulsive behaviors if they were high in positive urgency, over and above other facets of impulsive personality. Further, positive urgency is elevated in first degree relatives of those with schizophrenia, which may be a characteristic to target in treatment to prevent the development of schizophrenia in first degree relatives (99).

### Urgency and treatment outcomes

Recently, urgency has been found to influence and impede the effectiveness of substance use disorder treatments. Hershberger and colleagues (100) conducted a meta-analysis of studies reporting UPPS-P traits at the beginning of cognitive-behavioral treatment for substance use disorders and found that negative urgency (along with lack of planning) at treatment admission predicted poorer response at the end of treatment. This study also found that UPPS-P traits did not change markedly in treatment, necessitating a more directed approach to reducing these traits. In another study, positive urgency was related to increased (rather than decreased) alcohol use and problems following a text-based intervention for 21^st^ birthday drinking (101). Manasse and colleagues (102) similarly found that individuals with higher negative urgency at baseline experienced slower and less pronounced benefit from treatment. There is much more to understand concerning how and why urgency might impede or worsen the effects of treatment and how best to intervene to reduce the negative effects of urgency (see 103).

### One construct or two?

While there is strong evidence to suggest that negative urgency and positive urgency are two distinct constructs (10, 104–106), there is still debate about whether these two constructs are better understood as one tendency. Recently, there has been contradictory evidence supporting both sides of this debate. First, Cyders and Smith (104) tested multiple models of the UPPS-P and found that the model designating positive and negative urgency as one factor did not fit the data well, but that the model designating them as sub-factors under a broader urgency factor fit the data best. This was also supported in another study using a more diverse and representative sample (62). Another recent study examined alternative factor models of impulsivity within the UPPS-P to determine whether the five facets of the UPPS-P were independent from one another using confirmatory factor analysis and found that the five-factor model was supported (107). Additionally, Goh and colleagues (108) examined the UPPS-P model of impulsivity utilizing network analysis and found support for “five conceptually distinct and differentially related dimensions” indicating that positive and negative urgency are two distinct constructs.

However, it may be beneficial to conceptualize urgency as one construct. Support for this perspective comes from evidence that the two traits are highly correlated and may be theoretically indiscernible from one another (85, 91, 109, 110). Specifically, Billieux and colleagues (110) concluded that the two facets of urgency converge as a single cluster using item-based network analysis and that it may be more efficient to examine urgency as one construct. Additionally, the high correlation between negative and positive urgency, usually in the range of 0.6 to 0.8, suggests that these two traits may have more shared than distinct variance. In the end, it may be that the distinctiveness of the two traits may be difficult to establish, and it may depend on the sample in which they are measured, the outcomes under examination, and how the traits may cluster together in any particular dataset.

### Empirical brain correlates of urgency

Although still in relatively early stages, the empirical research concerning brain correlates of urgency has identified a number of key structures and circuits that contribute to cognitive control, emotions, and salience (see a review by 111). To date, the majority of these studies have primarily focused on negative urgency, rather than positive urgency. Greater dorsolateral prefrontal cortex activation was associated with negative urgency during a simple cognitive control task, indicating that those high in urgency may use greater cognitive resources in cognitive control tasks (112, 113). This may indicate that those high in urgency may use greater cognitive resources during cognitively demanding tasks, making it more difficult to engage in cognitive control (114). Increased right insula activation was related to negative urgency in a decision-making task in which the subject made a risky decision over a safe decision (115). Activation in the orbitofrontal cortex, which is associated with emotion-based learning and decision-making (see reviews by 116, 117), has been found to be significantly related to negative urgency in response to valenced mood images (118). Research has shown that negative urgency relates to increased activation in the left amygdala, a region involved in negative emotion processing, in response to negatively valenced images (118). Increased negative urgency has been linked to a smaller left ventral striatum and lower regional gray matter volume in the right temporal pole (119).

Studies that include positive urgency often report overlapping neuroanatomical correlates with negative urgency, indicating that emotional dispositions to rash action are implicated within the same regions, regardless of the valence of the emotion (see review by 111, 120). These shared neural correlates are indicated by high gray and white matter intraclass correlation analyses and a similarity of significant regions in linear mixed effect models (120). However, in elastic net analyses, positive urgency was better predicted by structural MRI than negative urgency, despite their overlapping neural correlates (120). Positive urgency has been implicated in greater left frontal asymmetry from the right anterior cingulate cortex, the medial frontal gyrus, and the right inferior frontal gyrus in EEG studies (121, 122). Positive urgency has also been shown to be related to decreased dopamine receptor availability in the bilateral nucleus accumbens, putamen, and caudate (123).

## Theories suggesting that emotions contribute to both adaptive and maladaptive decision-making: integrating Urgency Theory

Several theories model the capacity of emotions to be *both* adaptive and maladaptive for decision making, depending on contextual and situational factors. We review four such theories here that we believe provide viable, albeit different, approaches for integrating Urgency Theory into the broader emotion literature.

## Integration using model-based and model-free emotion regulation

Emotion regulation refers to one’s ability to cope, change, or respond to an emotional experience, and moves through a number of stages and processes, including selection of an emotional experience to modify, attention to a situation, cognitive appraisal of an emotional experience, and an attempt to modulate or respond to the situation (e.g., 124). Although much of the literature on emotion regulation has focused on the modification of negative emotions, some have highlighted the importance of regulating positive emotions as well (e.g., 125), in that maintaining or increasing positive emotional experiences may underlie resilience. Emotion regulation theories propose that emotions that are regulated can be adaptive, whereas emotions that are not regulated may lead to more maladaptive coping (which would be referred to as emotion dysregulation; e.g., 126, 127). For example, a person experiencing depression might be motivated to seek out help, which would be an adaptive emotion-guided response. However, ongoing depression that does not fuel help- or treatment-seeking can be quite maladaptive; such maladaptive responses might be driven, in part, by a lack of awareness of emotions (e.g., 128).

There are several emotion regulation strategies that have been linked to positive outcomes. For example, emotion reappraisal (i.e., “modifying the emotional meaning and impact of a situation that elicits emotion,” 129) of both positive and negative emotions is associated with fewer depressive symptoms, greater self-esteem, life satisfaction, and overall wellbeing (129). Reappraisal has also been shown to decrease negative emotions and increase positive emotions in lab paradigms and when using self-report measures (129, 130). Additionally, individuals that utilize reappraisal report having closer relationships due to the increased likelihood of them sharing their emotions (129, 131), illustrating that emotion reappraisal can be adaptive and, in turn, lead to positive outcomes. MacDonald and colleagues (132) found that rapid improvement in emotion regulation strategies significantly increased posttreatment binge/purge abstinence, decreased depression symptoms, and decreased eating disorder-related cognitive psychopathology at posttreatment for individuals diagnosed with Bulimia Nervosa or Purging Disorder. A creative laboratory-based experiment (133) sought to determine the contexts that influence the adaptiveness of negative emotions, finding that negative emotions linked to context (e.g., being sad in the face of a family member passing away) produce more adaptive responses and were associated with better psychological health and adjustment. Thus, this suggests that negative emotions that match the current needs of an individual and that are successfully regulated are and can be adaptive, whereas those that do not match the current needs or are not regulated can be maladaptive.

Etkin and colleagues (134) propose that the decision to engage in emotion regulation is linked to the predicted outcomes of that regulation – in this way, emotion regulation strategies that are linked to a desired outcome are engaged in, whereas those that are associated with costs are avoided. There is continual updating of these assessments over time, and value predictions (i.e., predictions one makes about the relative cost-benefit of an emotion regulation strategy) that are discrepant with one’s current experiences are considered “prediction errors” (134). The authors explain this within the context of model-based and model-free control: In model-free control, one makes a decision based on one’s current assessment of prediction errors – i.e., the discrepancy between one’s desired state and their current state – which is efficient but not flexible. In contrast, in model-based control, one makes a decision by applying prior knowledge, which is less efficient, but more optimal. The authors propose different underlying circuits related to model-based and model-free emotion regulation, such that model-free emotion regulation may be driven by ventral anterior cingulate cortex and ventromedial prefrontal cortex interactions with limbic-emotional circuits, whereas model-based emotion regulation is governed by frontoparietal regions (e.g., ventral lateral prefrontal cortex, dorsolateral prefrontal cortex, parietal cortex, and supplementary motor areas) interacting with limbic-emotional circuits (134).

### Empirical evidence integrating urgency

The literature overall has supported the idea that urgency and emotion (dys)regulation are related (92, 135–143). Some have suggested that urgency is driven, in part, by low levels of emotion regulation (64). Others have found negative relationships between negative urgency and the use of adaptive emotion regulation strategies (112) and that differing levels of urgency within individuals results in different kinds of emotion regulation strategies (144). In addition, research in the brain shows overlap in regions implicated in emotion regulation and urgency. A recent review documented that emotion regulation is underpinned by the lateral prefrontal cortex and the amygdala (145), regions that overlapped with cognitive control and emotion regions related to urgency (see review by 111).

There is one only empirical study directly testing the idea of urgency as model-free emotion regulation. Jara-Rizzo and colleagues (146) conceptualized negative urgency as a sign of poor emotion regulation (also supported by 112), and found that negative urgency is related to emotional suppression, but not reappraisal. The authors suggest that negative urgency results from model-free emotion dysregulation (146), such that model-based emotion regulation may result in emotions facilitating adaptive decision-making, whereas model-free emotion regulation may result in emotions producing more maladaptive decision-making. Such a proposal is viable, based on previous work showing a relationship between brain activity in the ventromedial prefrontal cortex in response to alcohol cues and both negative urgency (e.g., 118) and model-free emotion regulation (134). We see the model-based and model-free framework as an interesting way to guide future investigations aimed at better integrating Urgency Theory with models of emotion regulation and adaptive decision-making.

## The mood maintenance hypothesis and the affect infusion model: integration using integral and incidental emotions

Two contrasting theories suggest opposite effects across positively and negatively valenced states and are considered jointly here. The Mood Maintenance Hypothesis suggests that positive mood improves decision-making, producing less risky decisions, whereas negative moods lead to less adaptive decision-making and more risk-taking, in the pursuit of obtaining a reward and bettering one’s mood (147, 148). In contrast, the Affect Infusion Model suggests that positive affect produces less adaptive decision-making, through attending to the positive cues in the environment which may make one overly optimistic about an outcome; whereas negative affect leads to more adaptive decision-making, through attending to the negative cues in the environment and acting in ways to avoid subsequent negative outcomes (149). An interesting study by Grable and Roszkowski (150) found more support for the Affect Infusion model, suggesting that negative affect may be more linked with adaptive decision-making and that positive affect may be more linked with maladaptive decision-making.

Lerner and colleagues (35) propose how integral and incidental emotions may explain how similar emotional valence states may produce differential effects on decision-making while different emotional valence states may produce similar effects on decision-making. Integral emotions are emotions that arise from the situation or decision at hand, and are thought to underlie decision-making in models, such as the Somatic Marker Hypothesis (151). Lerner and colleagues (35) summarize the research on integral emotions as largely providing evidence that emotions benefit and improve decision-making (although there is some evidence that integral emotions can bias decision-making when they do not reflect reality and that in these cases, they can override more rational courses of action; see a review by 152). Incidental emotions, on the other hand, are emotions that carry over from another situation and influence later decisions (35), a process known as the carryover of incidental emotion (e.g., 153, 154). Lerner and colleagues (35) summarize the research on incidental emotions as largely providing evidence for the biasing effects of incidental emotions, a process by which a more diffuse mood continues to bias decision-making even outside of the primary event or situation which activated the initial emotion response.

### Empirical evidence integrating urgency

There is no existing empirical evidence directly linking urgency with integral and incidental emotions. However, the idea of integral and incidental models may guide attempts to integrate Urgency Theory and adaptive emotion-guided decision-making. It’s possible that adaptive emotion-guided decision-making is influenced largely by integral emotions, whereas more maladaptive, urgency-like decision-making is influenced largely by incidental emotions. Phelps and colleagues (34) suggest that incidental emotion effects on decision-making may be driven by impaired function of the PFC, or shifting neural processes in regions, such as the orbitofrontal cortex, that assess subjective value. Although there is more work to do in this regard, these underlying brain correlates seem to overlap with regions related to negative urgency (e.g., see 118). This may suggest viability of viewing urgency as a mechanism underlying the detrimental effects of incidental emotions on decision-making. Evidence from neuroimaging work shows that the amygdala may underlie task-independent processing of emotions, whereas the ventromedial prefrontal and somatosensory cortices may be involved in more direct processing of emotions (155). Urgency shows relationships with amygdala functioning (118), indirectly supporting the integration of urgency with incidental processing. However, evidence also links urgency with the ventromedial prefrontal cortex (118), suggesting that urgency may not be reflected by the distinction between incidental and integral processing.

## Integration using reflexive responsivity to emotions

Carver and colleagues (156) proposed a model wherein emotions are responded to thoughtfully, which would be associated with more adaptive outcomes, or reflexively, which would be associated with less adaptive outcomes. Reflexive responses can be rash action (like in the case of urgency) or rash inaction (e.g., not seeking out treatment for depression, as in the example above) (156). Thus, emotions that are responded to thoughtfully produce adaptive responses. Carver’s theory can serve to integrate across Urgency Theory and theories that highlight the adaptiveness of emotions for decision-making. Adaptive or maladaptive responses to emotions might be better subsumed as reflexive or thoughtful behavioral action or inaction (and reflexive and thoughtful responses can be either adaptive or maladaptive in nature). In this model, adaptive emotion-guided decision-making would be represented as thoughtful responses, whereas urgency can be better understood as a marker of reflexive responses to emotions (whether by action or inaction; as supported by 157).

### Empirical evidence integrating urgency

There are mixed empirical data to suggest that urgency and reflexive responses to emotions are similar constructs. Smith and colleagues (157) found that urgency appears to be a marker of reflexive responses to emotions as proposed by the Carver model, in that urgency can relate to both rash action and rash inaction; the common factor here appears to be the focus on alleviation of distress or focusing on the immediate emotion rather than the underlying cause of the emotion itself (157). A 2018 study found that negative urgency was associated with using more reflexive emotion regulation strategies, providing support for Smith et al.’s findings (144). However, a recent study by Sperry and colleagues (158) utilizing Ecological Momentary Assessment (EMA) to examine the link between affect and impulsivity did not find an association between internalizing/externalizing psychopathology and momentary links between affect and impulsivity, suggesting that urgency and reflexive responses to emotion may not be the same thing. There is much more research to be done to determine the validity of this option as explaining the integration of these theories, but it is nonetheless a viable and interesting future avenue of investigation.

## Integration using individual difference theory

Individual difference theories posit that there are variations across people on numerous factors that drive behavior, including personality, intelligence, and emotionality. These differences can be driven by temperament, learning, or even underlying biological, genetic, or neural underpinnings (e.g., 159). Urgency was proposed as an individual difference tendency (10), suggesting that Urgency Theory is not universal, but rather only applies to a subset of the population.

### Empirical evidence integrating urgency

There is much literature to support the idea that urgency is an individual difference trait (see a review by 160). However, there is also evidence that works against the idea that urgency is an individual difference variable, including that urgency can change in response to treatment or intervention (e.g., 100, 161). This undermines the idea that it is a stable, trait-like construct (e.g., 162, 163), although some suggest that individual differences can change over time (e.g., 164). Thus, although the fact that urgency can change doesn’t completely rule out the idea that it is a relatively stable individual difference variable, it is a consideration. Although the field overwhelmingly models urgency as a trait, other approaches have treated urgency as a state-like construct that can be manipulated in the laboratory (see a review by 165).

## Conclusion and future directions

In this conceptual review, we conclude that Urgency Theory can be integrated into broader emotion models that include both adaptive and maladaptive effects of emotions on decision-making. We highlight four, not mutually exclusive, possibilities: urgency as a function of model-free emotion (dys)regulation (134); urgency reflecting a reliance on incidental, rather than integral, emotions (as reviewed by 35); urgency as a reflexive, rather than purposeful, response to emotions (156); or urgency as an individual difference tendency that may only apply to a subset of the population.

Although all these possibilities are viable, there is little empirical evidence to inform which, if any, of the theories proposed here best reflect truth. There is no research directly comparing or integrating these constructs into one model; filling this gap is an important future research direction. Some evidence testing these theories is better well-developed than others, suggesting greater viability for some approaches than others; however, other theories are under-researched, suggesting that they may still be viable but require additional empirical study. Measuring these constructs using multiple methods, including self-report, behavioral responding, and neuroimaging, as well as examining the inter-relations among these constructs by testing multiple factor models, would have the most impact in filling this gap. Identifying how and why urgency imparts risk can facilitate development and testing of effective interventions to decrease maladaptive risk-taking, identification of those at risk for maladaptive risk-taking, and matching of interventions to those at risk. Whether or not Urgency can be validly integrated into the above models has impact for how best to intervene.

To date, the most supported integrative theory is that urgency is an individual difference tendency (see a review by 160). This is also the most well-researched theory. Although overwhelmingly supported, studies exist modeling urgency as a state-like behavior, which may undermine the trait-like view of urgency. In all, it appears that urgency is best conceptualized as a trait, where individuals exist on a continuum (i.e., some have high levels, some low levels, and some in the middle), where the higher the trait, the more the individual engages in maladaptive action while experiencing emotional states. Evidence in the treatment literature suggest that urgency changes very little during treatment (100, 161), supporting the idea that urgency is trait-like and may be difficult to change, suggesting that targeting urgency in a treatment setting may not be a viable approach. Clinically, if urgency is best represented as an individual difference tendency, it would be most effective to identify those high in urgency to receive targeted interventions to reduce urgency-related risk-taking and maladaptive behaviors.

The second best supported integrative theory is urgency model-free emotion dysregulation, with the field generally conceptualizing urgency as associated with higher levels of emotion dysregulation (although the exact mechanism of this relationship is not yet well known). More work is needed to better understand if urgency and emotion dysregulation are the same thing, or if they are two separate, though related, constructs that influence one another or are related due to the presence of a third factor. There is one only empirical study directly testing the idea of urgency as model-free emotion regulation; this study supported the idea of urgency as model-free emotion (146). Replication of this finding is important. Clinically, if urgency is a function of model-free emotion (dys)regulation (134), this would suggest that urgent behaviors may be reduced through a more purposeful use of previous learning structure in the selection of emotion regulation strategy rather than relying on reacting to one’s current state. One study (92) applied emotion regulation skills training and found reductions in both urgency and emotion dysregulation, supporting this integration and intervention approach.

The final two theories have less empirical support. The data connecting urgency to a reflexive response to emotions is mixed. Clinically, if urgency reflects a reflexive response to emotions, this would suggest that slowing down and making the decision-making process explicit might be a viable way to interrupt urgent behaviors. However, more direct empirical tests of this integration are necessary to determine if this approach might be effective. The mixed evidence may suggest that there are individual differences in urgency, further supporting the individual difference integration. Finally, to date, no empirical studies have directly tested the integration of urgency with incidental emotions, so a conclusion cannot be made as to the viability of this approach at this time. This integration is supported by theory and overlapping brain regions, but direct empirical tests are needed to determine if this approach is feasible. Clinically, if urgency reflects a reliance on incidental emotions, this would suggest the viability of reducing urgent behaviors through reorienting one’s attention away from unrelated emotional experiences and toward ones that are applicable to one’s current state. This would need to be tested before clinical application.

As this was a conceptual review, other potential ways to integrate Urgency Theory may exist. We hope that this review catalyzes future empirical research aimed at understanding how best to integrate urgency into these (or other) theories. Although all four options are viable based on existing empirical evidence and theory, individual difference and model-free emotion (dys)regulation have the most support. Importantly, the other two options are less well-researched. Direct tests comparing these integrations would be necessary to determine the most accurate way to integrate urgency with existing emotion theories. We believe that this research can identify key mechanisms underlying urgency and help inform how best to target and modify urgency to reduce its negative effects across numerous maladaptive behaviors and clinical disorders.

## Author contributions

LF-F: Conceptualization, Funding acquisition, Writing – original draft, Writing – review & editing. CP: Writing – original draft, Writing – review & editing. MC: Conceptualization, Funding acquisition, Supervision, Writing – original draft, Writing – review & editing.
